# Supplementary material for: The Effects of Virtual Reality Interventions on Motor Function Rehabilitation in Lower-Limb Amputees: A Systematic Review and Metanalysis
Source: Bioengineering (Basel). 2025 Oct 28;12(11):1170. doi: 10.3390/bioengineering12111170 (PMC12649739; doi:10.3390/bioengineering12111170)
Supplement: Supplementary file 1 [file bioengineering-12-01170-s001.zip › bioengineering supplementary S2.pdf]

Supplementary file. Search strategy.

Database	Search strategy
PubMed	(("Virtual Reality"[MeSH Terms] OR "Virtual Reality Exposure Therapy"[MeSH Terms] OR "Exergaming"[MeSH Terms]) AND ("amputation, surgical"[MeSH Terms] OR "amputation, traumatic"[MeSH Terms] OR "Amputation Stumps"[MeSH Terms]))
Scopus	(Amput* OR Amputate OR Amputation OR "Amputation Procedure, Surgical" OR "Amputation Procedures, Surgical" OR "Amputation Stump" OR "Amputation, Multiple, Surgical" OR "Amputation, Surgical Multiple" OR Amputations OR "Amputations, Surgical" OR "Amputations, Surgical Multiple" OR "Amputations, Traumatic" OR Amputee OR Disarticulat* OR Disarticulation OR Exarticulat* OR "Limb loss" OR "Lower limb amputation" OR "Multiple Amputation, Surgical" OR "Multiple Amputations, Surgical" OR "Procedure, Surgical Amputation" OR "Procedures, Surgical Amputation" OR "Prosthetic limb" OR "Stump, Amputation" OR "Surgical Amputation" OR "Surgical Amputation Procedure" OR "Surgical Amputation Procedures" OR "Surgical Amputations" OR "Surgical Multiple Amputation" OR "Surgical Multiple Amputations" OR "Traumatic Amputation" OR "Traumatic Amputations") AND ("Active Video Gaming" OR Active-Video OR "Active-Video Gamings" OR "Augmented reality" OR "Computer-simulated reality" OR "Educational Virtual Reality" OR Exergame OR Exergames OR Exergamings OR Exergam* OR "Exercises, Virtual Reality" OR "Exercise, Virtual Reality" OR Gaming OR "Gaming, Active-Video" OR "Gamings, Active-Video" OR "Head mounted displa*" OR Headmounteddevic OR "Immersive VR" OR "Instructional Virtual Reality" OR Kinapsys OR Kinect OR "Medical technology" OR "Mixed reality" OR "Non-immersive VR" OR "Reality Therapies, Virtual" OR "Reality Therapy, Virtual" OR "Serious gam" OR Smartglass OR "Therapies, Virtual Reality" OR "Therapy, Virtual Reality" OR "Video game" OR "Virtual environment" OR "Virtual reality-enhanced" OR "Virtual Reality (VR) technology" OR "Virtual Reality Exercises" OR "Virtual Reality exposure" OR "Virtual Reality Immersion Therapy" OR "Virtual Reality Therapies" OR "Virtual Reality therapy" OR Wii)
Web of Science	(Amput* OR Amputate OR Amputation OR Amputations OR "Amputations, Surgical" OR "Amputations, Traumatic" OR Amputee OR "Limb loss" OR "Lower limb amputation" OR Disarticulat* OR Disarticulation OR "Amputation Stump" OR "Prosthetic limb" OR "Surgical Amputation" OR "Surgical Amputation Procedure" OR "Surgical Amputations" OR "Surgical Multiple Amputations" OR "Traumatic Amputation" OR "Multiple Amputations, Surgical" OR "Amputation Procedure, Surgical" OR "Stump, Amputation" OR "Amputation, Surgical Multiple" OR "Surgical Multiple Amputation") AND ("Active Video Gaming" OR "Augmented reality" OR "Computer-simulated reality" OR "Educational Virtual Reality" OR Exergame OR Exergames OR Exergam* OR "Exercise, Virtual Reality" OR "Exercises, Virtual Reality" OR Gaming OR

	Kinect OR Wii OR "Immersive VR" OR "Mixed reality" OR "Non-immersive VR" OR "Virtual environment" OR "Virtual reality-enhanced" OR "Virtual Reality (VR) technology" OR "Virtual Reality Exercises" OR "Virtual Reality Immersion Therapy" OR "Virtual Reality Therapies" OR "Virtual Reality therapy" OR "Virtual Reality exposure" OR "Instructional Virtual Reality" OR "Therapy, Virtual Reality")
